# On the Feeding of Infants

**Published:** 1892-04-23

**Authors:** 


### The Practitioner's Mirror and Retrospect.

[*The Editor will be glad to receive offers of co-operation and contributions from members of the profession. All letters should be addressed to* The Editor, The Lodge, Porchester Square, London, W.]

### MEDICINE.

### ON THE FEEDING OF INFANTS.

From many causes it may happen that a mother is unable to nurse her child. When that is the case, and the child cannot have a wet nurse, it must be artifically fed. The difficulty then confronts us of deciding what kind of food is the most suitable. We recognise that the food must be liquid, and must approximate as closely as possible in chemical and physical properties to human milk.

The proteid constituent of milk consists partly of casein, partly of lact-albumen. On arrival in the stomach, the casein is precipitated, by the action of the rennet ferment, as tyrein (or cheese), and subsequently this tyrein, together with the lact-albumen, are acted on by the pepsin, and converted into albumoses and peptones, which are absorbed. Now a peculiar characteristic of the casein of human milk is that it is precipitated by the rennet ferment in a fine flocculent form, not in massive curds. It can thus be most easily acted on by the pepsin. This property is not shared by any of the commonly employed kinds of milk, with exception of that of the ass. For instance, the casein of cow's milk is precipitated as a massive clot, which takes a much longer time in being digested than does the casein of human milk, and so is much more liable meanwhile to irritate the stomach, and to decompose and set up vomiting and diarrhœa.

Secondly, it is to be observed that the carbo-hydrate is in the form of milk sugar, which can be absorbed without previous digestion. Correspondingly, it is found that in the infant neither the salivary glands nor pancreas produce those ferments, ptyalin and pancreatic amylolytic ferment, which convert starches into sugar. Very little saliva is secreted until the teeth begin to appear, and the pancreas does not produce its amylolytic ferment in large quantities until nearly the end of the first year.

It follows from this that the carbo-hydrate in an infant's food must be in the form of sugar, not of starch. This little appreciated fact immediately leads us to condemn all those farinaceous foods in which the carbo-hydrates are present as starch; to give these to infants is not merely to give them something they cannot digest, but what thereby causes indigestion, with its train of results.

A third most important quality of human milk is that the infant drinks it quite fresh and absolutely free from the slightest trace of decomposition. Milk is about one of the best culture media for bacteria we know, and so unless hermetically sealed up in a perfectly clean vessel, soon absorbs micro-organisms from the air and decomposes. The results of this as a food are gastro-intestinal catarrh of varying degrees of severity, worse, as might be expected, in the hot summer months.

Milk identical with fresh human milk is not produced from any other animal; we must, then, take the milk of some lower animal and render it as like as possible to human milk. We are in the great majority of cases restricted to cow's milk. The other kinds occasionally used may first shortly be dimissed. Asses' milk has the same physical properties, as regards its casein, as human milk, but it can rarely be obtained, and if it can, is but a weak solution, its composition being proteids, 1·7 per cent.; fats, 1·4 per cent.; carbo-hydrates, 6·4 per cent.; so that after the first month the child cannot drink enough to get the solids it requires. Goats' milk is richer than cows', but its casein coagulates in massive clots, so that it is even more indigestible than cows' milk.

Regarding cow's milk, it may be observed that there is not the slightest advantage in having the milk from one cow only, for this is much more likely to vary than that got by mixing the milk of several. Further, unless the parent possesses three acres and a cow, it is most improbable that he really gets, if he asks for it, the milk from one cow only.

Having obtained the milk, the first thing is to boil it; this kills any micro-organisms that have got into it. It should then be put away in a perfectly clean, covered vessel in a cool place until wanted. This interval should never exceed twelve hours, for the milk will, though a little less readily than before, soon begin to decompose.

Now, cows' milk has not the same percentage composition as human milk, the percentage of proteid and fat being greater as the following table shows:—

| | Cows' Milk. | Human Milk. | 1 part water, 2 parts Cows' Milk. |
|---|---|---|---|
| Proteids | 5·4 per cent. | 3·5 per cent. | 3·6 per cent. |
| Fats | 4·3 „ „ | 3·0 „ „ | 2·9 „ „ |
| Sugar | 4·0 „ „ | 4·0 „ „ | 2·7 „ „ |

It must, therefore, be diluted. If we do this in the proportion of two parts of milk to one part of water we have a liquid in which the percentage of proteids and fats is about the same as in human milk, but in which the sugar is slightly deficient.

The difficulty, however, remains that the casein still comes down in massive clots on the addition of rennet. This can to a large extent be got over by employing some mucilaginous solution instead of plain water for diluting. The best, perhaps, to use is barley water, made by boiling a pint of water with a tablespoonful of pearl barley and straining. This acts, probably mechanically, on the casein, which is precipated in very much smaller masses.

Various other solutions have been proposed instead of the barley water, with which to dilute the milk, the commonest, perhaps, being lime water and a 5 per cent. solution of sodium carbonate. Both of these are effectual, but for other reasons objectionable, the former insomuch as it is constipating, the latter because its continual ingestion is in the long run harmful.

It would appear, then, that theoretically a most satisfactory food is boiled fresh cows' milk, diluted with one-half its volume of barley water, and slightly sweetened—for this, as we have seen, approaches fairly closely, in chemical and physical properties, to human milk. And practically we have found that for the very great majority of infants this mixture does very well; they neither vomit nor have diarrhœa nor get rickets, but steadily increase in size and weight.

Regarding condensed milk and other foods for infants that have been proposed, we will say something in a subsequent paper.

